# Correction: Spatial and temporal correlations in human cortex are inherently linked and predicted by functional hierarchy, vigilance state as well as antiepileptic drug load

**DOI:** 10.1371/journal.pcbi.1011094

**Published:** 2023-04-27

**Authors:** Paul Manuel Müller, Christian Meisel

In the Funding statement, the grant number from the funder NeuroCure Cluster of Excellence is listed incorrectly. The correct grant number is: EXC-2049-390688087.
