# Supplementary figures and images for: Spatial and temporal correlations in human cortex are inherently linked and predicted by functional hierarchy, vigilance state as well as antiepileptic drug load
Source: PLoS Comput Biol. 2023 Mar 3;19(3):e1010919. doi: 10.1371/journal.pcbi.1010919 (PMC10027224; doi:10.1371/journal.pcbi.1010919)

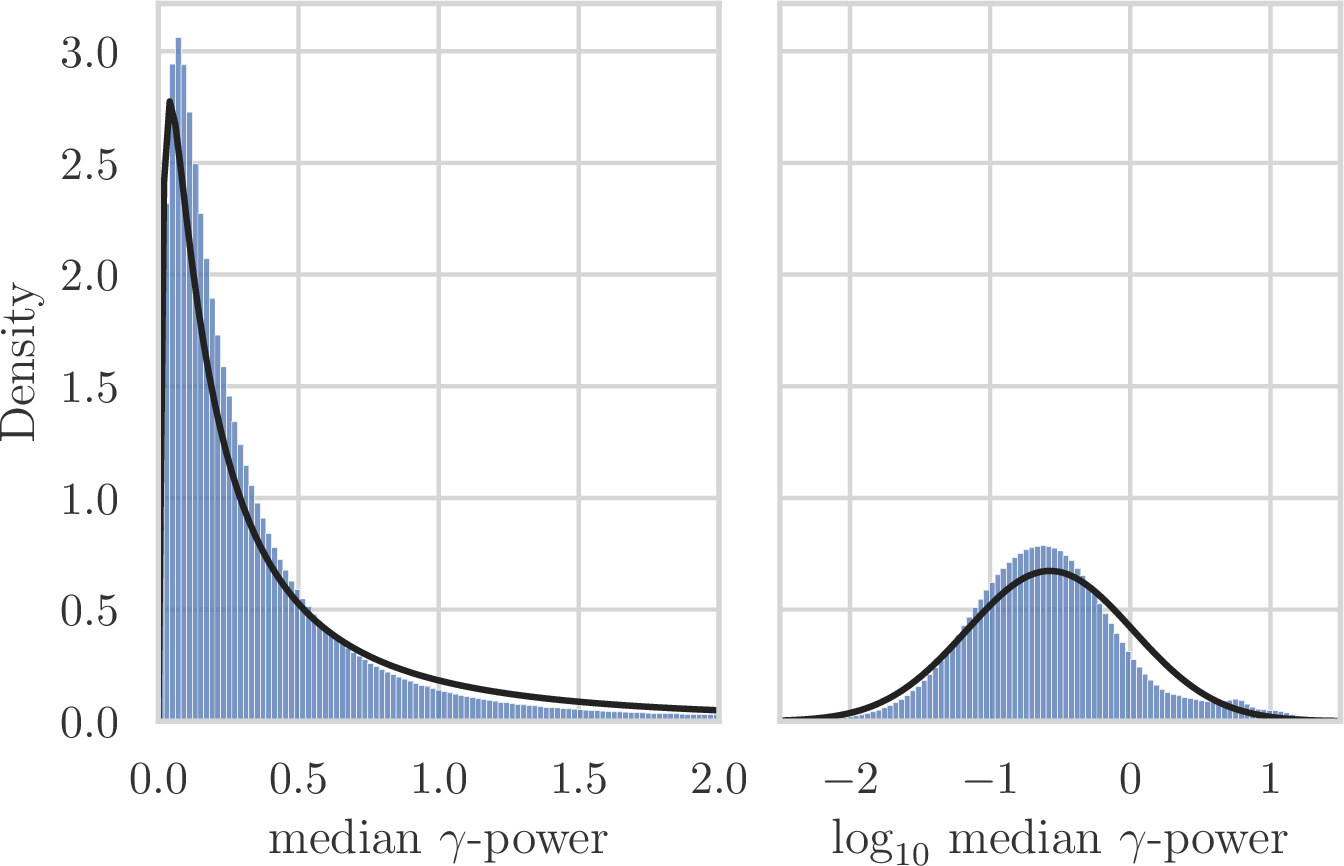

Supplement: S1 Fig — Left: Histogram of the median broadband γ-power. Right: Same data transformed by applying the logarithm with base 10. Black lines in both panels show a fitted log-normal distribution. (TIF) [file pcbi.1010919.s004.tif]

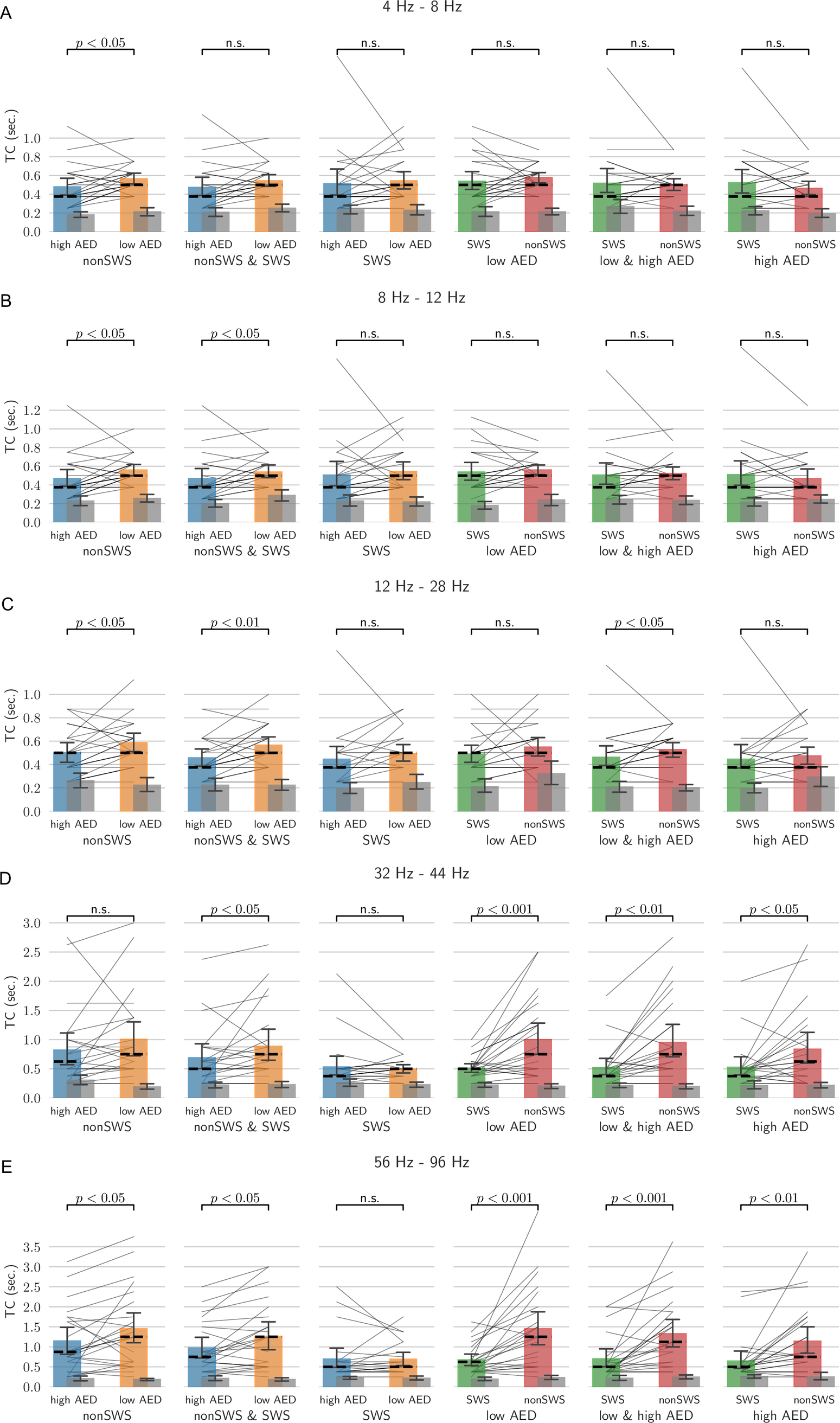

Supplement: S2 Fig — In each side-by-side plot only one state is changed, either the drug load or the sleep stage. (TIF) [file pcbi.1010919.s005.tif]

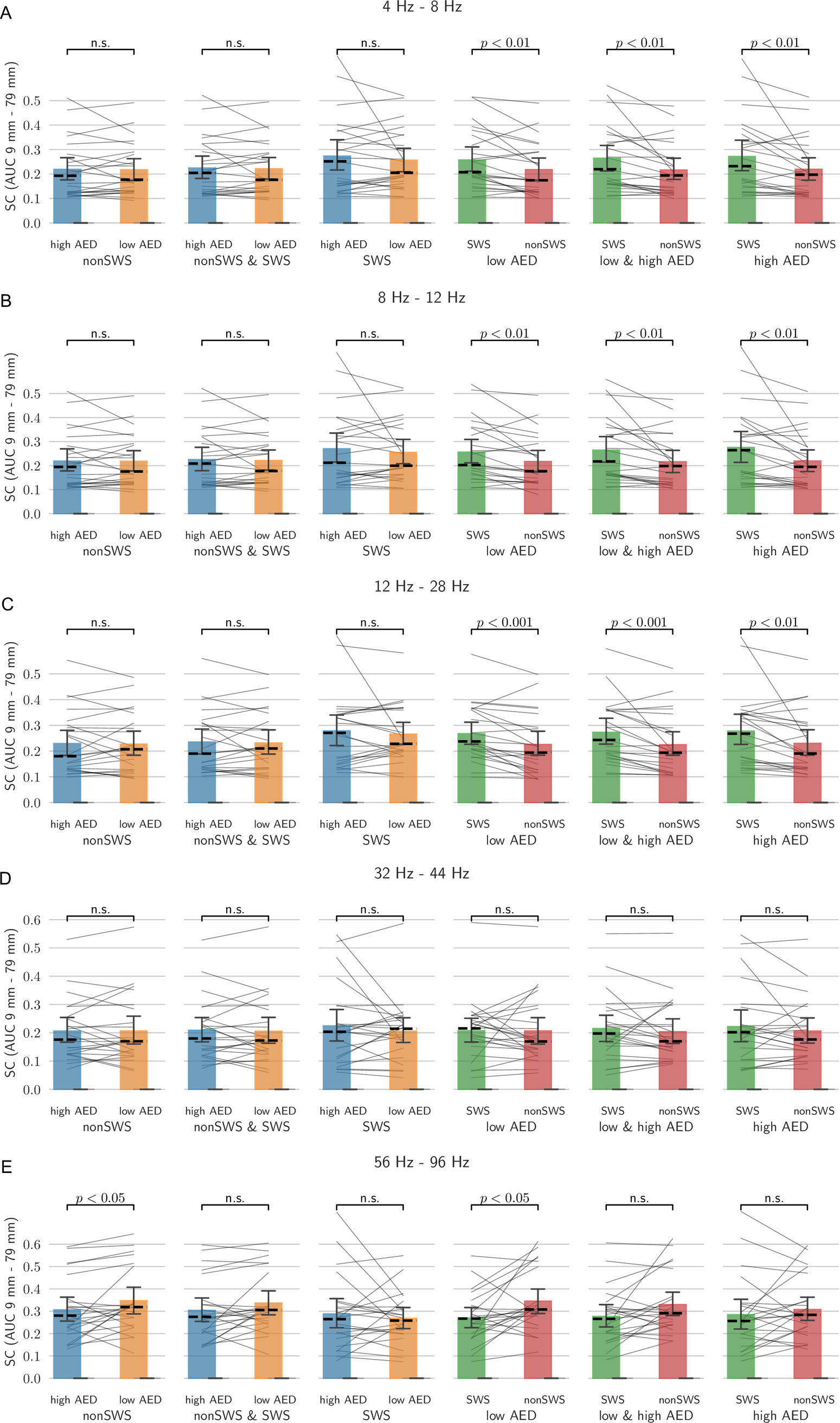

Supplement: S3 Fig — In each side-by-side plot only one state is changed, either the drug load or the sleep stage. (TIF) [file pcbi.1010919.s006.tif]

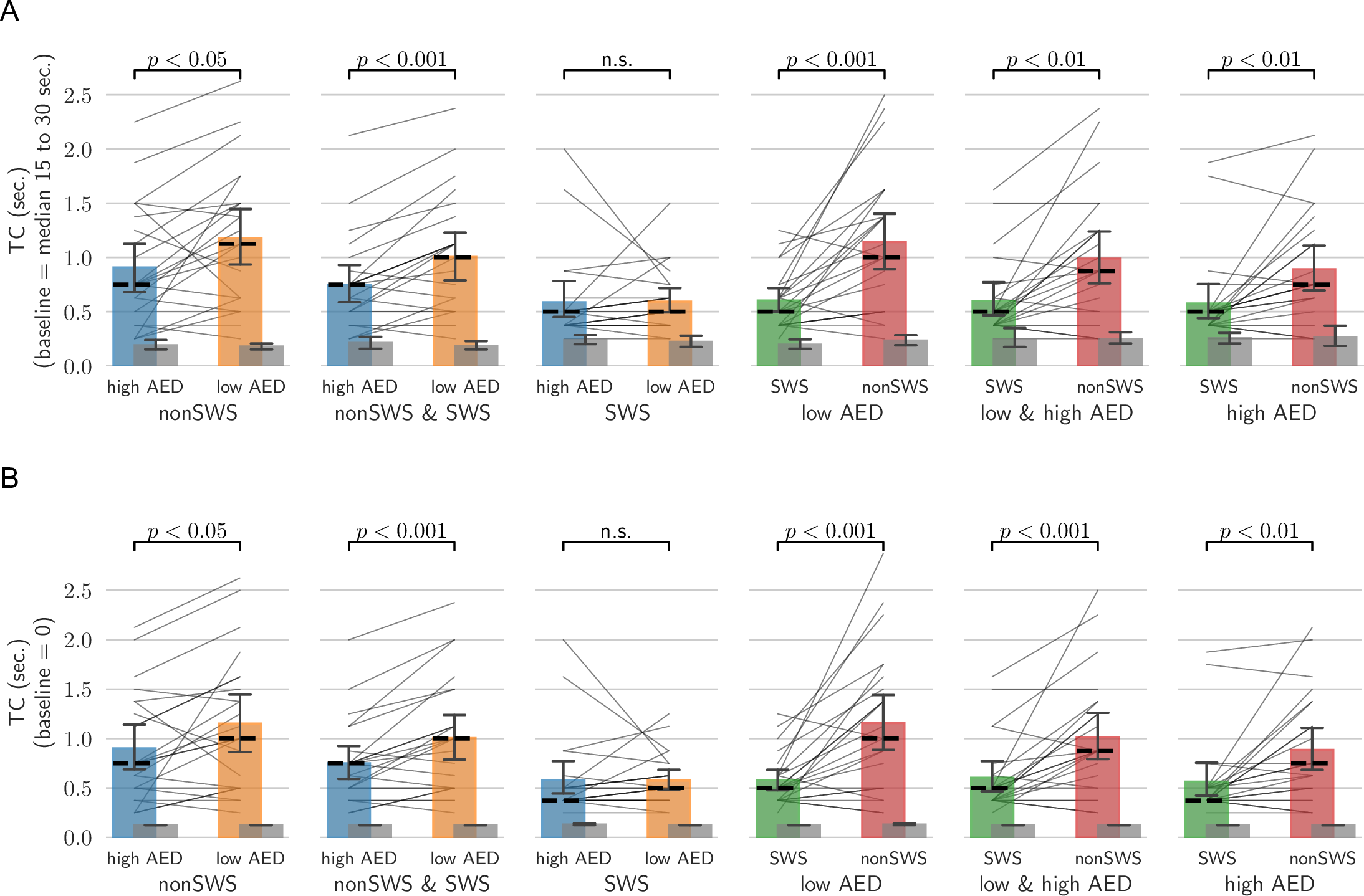

Supplement: S4 Fig — TC calculated from broadband γ-power for A the baseline defined as the median value between 15 and 30 seconds and B the baseline set to 0 for the calculation of the half maximum value. Results are quantitively the same as for the definition used in the main manuscript (baseline at the median value between 40 and 60 seconds, compare panel E in S2 Fig). (TIF) [file pcbi.1010919.s007.tif]

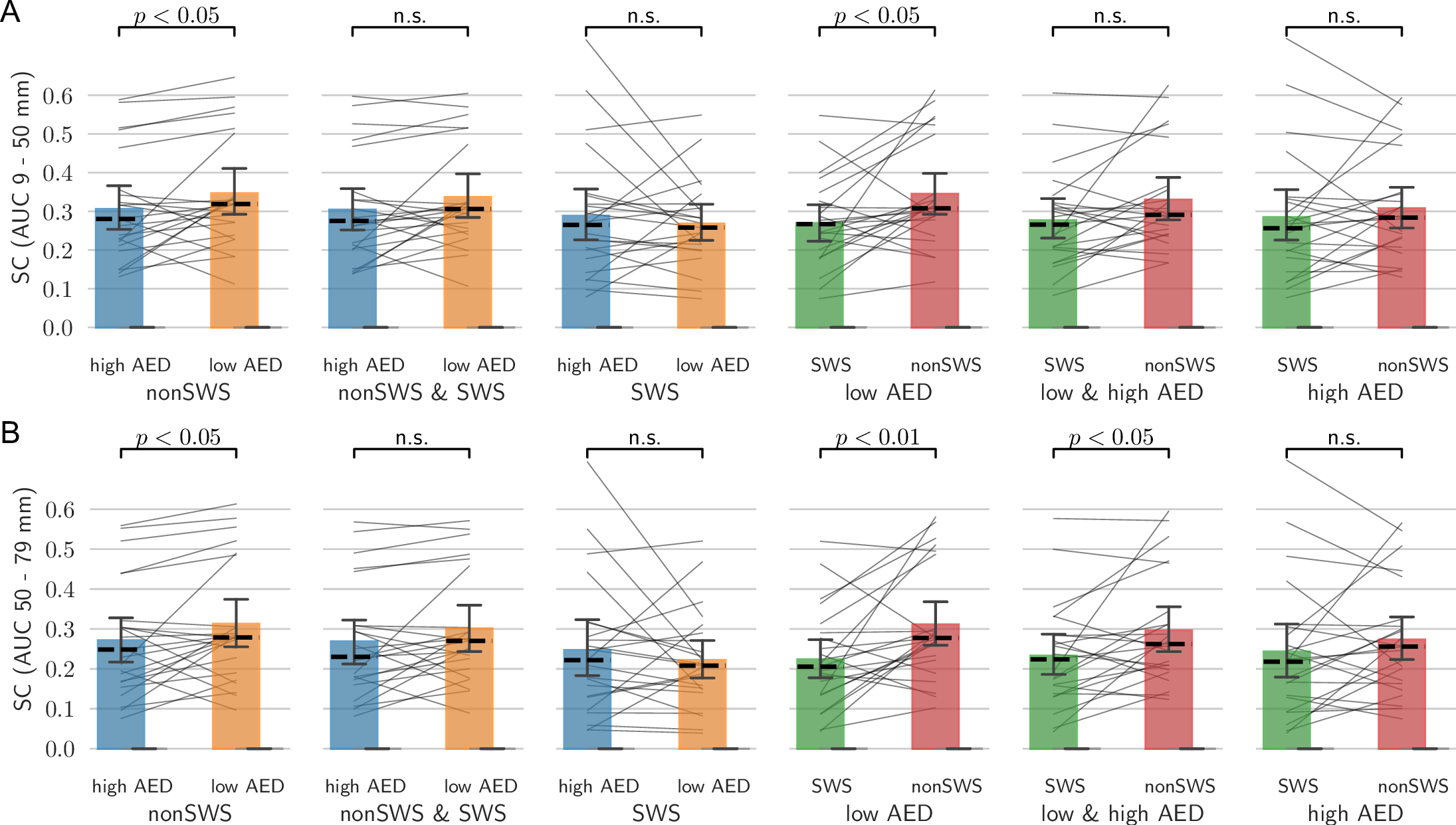

Supplement: S5 Fig — SC calculated from broadband γ-power defined as the area under the cross-correlation function between A 9 and 50 mm and B 50 and 79 mm. Results are quantitively the same as for the definition used in the main manuscript (area under the cross-correlation function between 9 and 79 mm, compare panel E in S3 Fig). (TIF) [file pcbi.1010919.s008.tif]

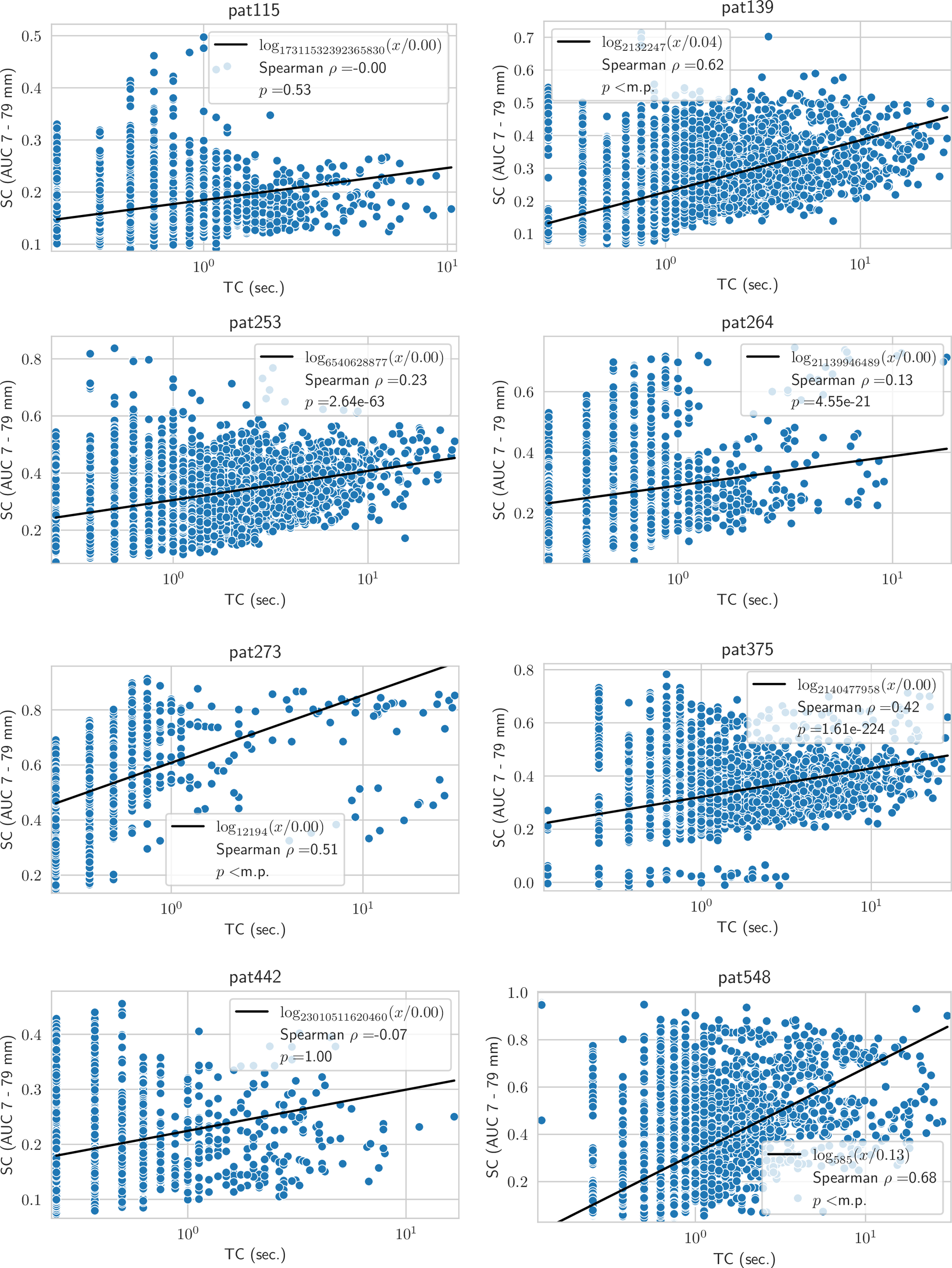

Supplement: S6 Fig — (m.p. = machine precision). (TIF) [file pcbi.1010919.s009.tif]

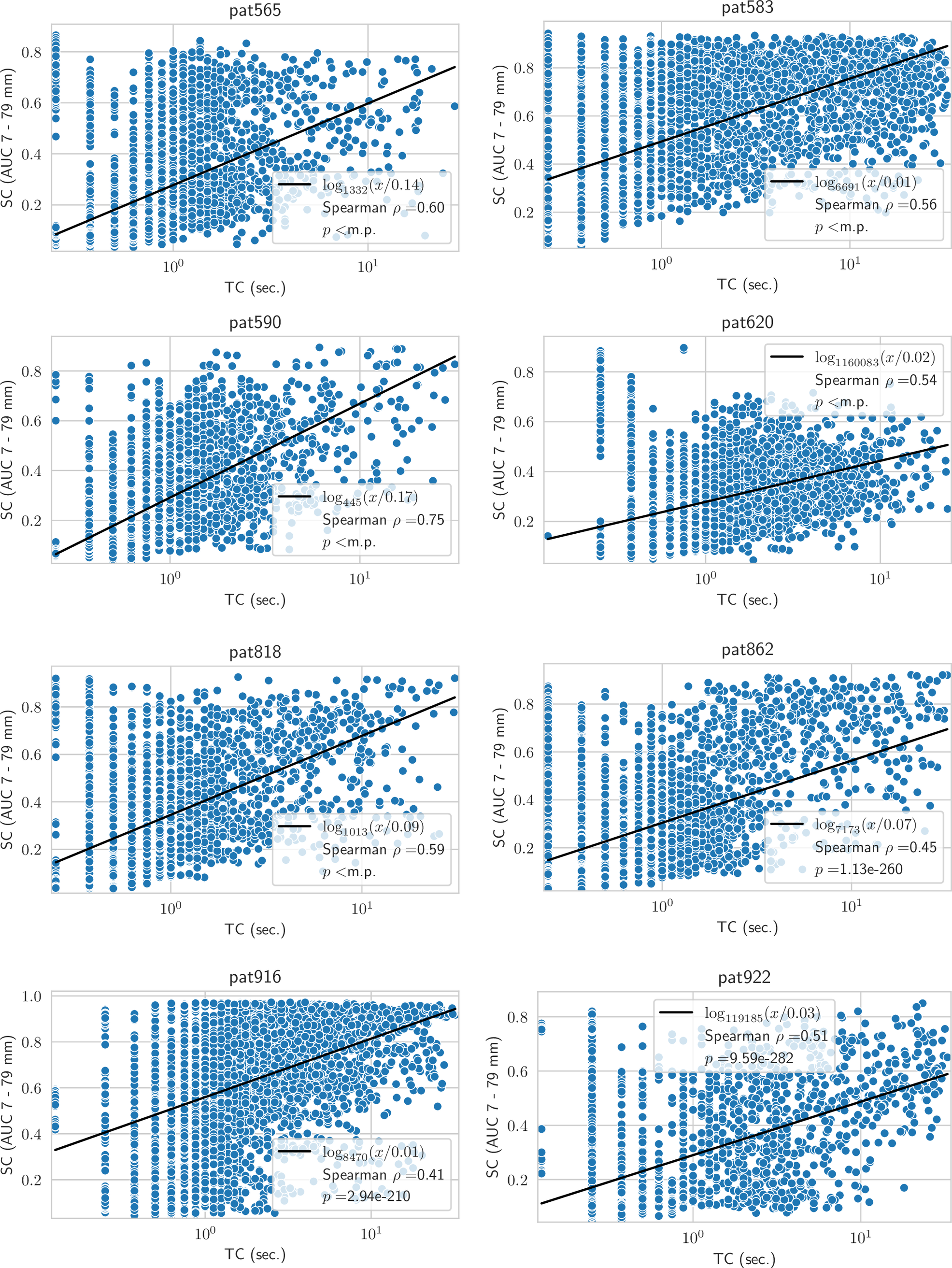

Supplement: S7 Fig — (m.p = machine precision). (TIF) [file pcbi.1010919.s010.tif]

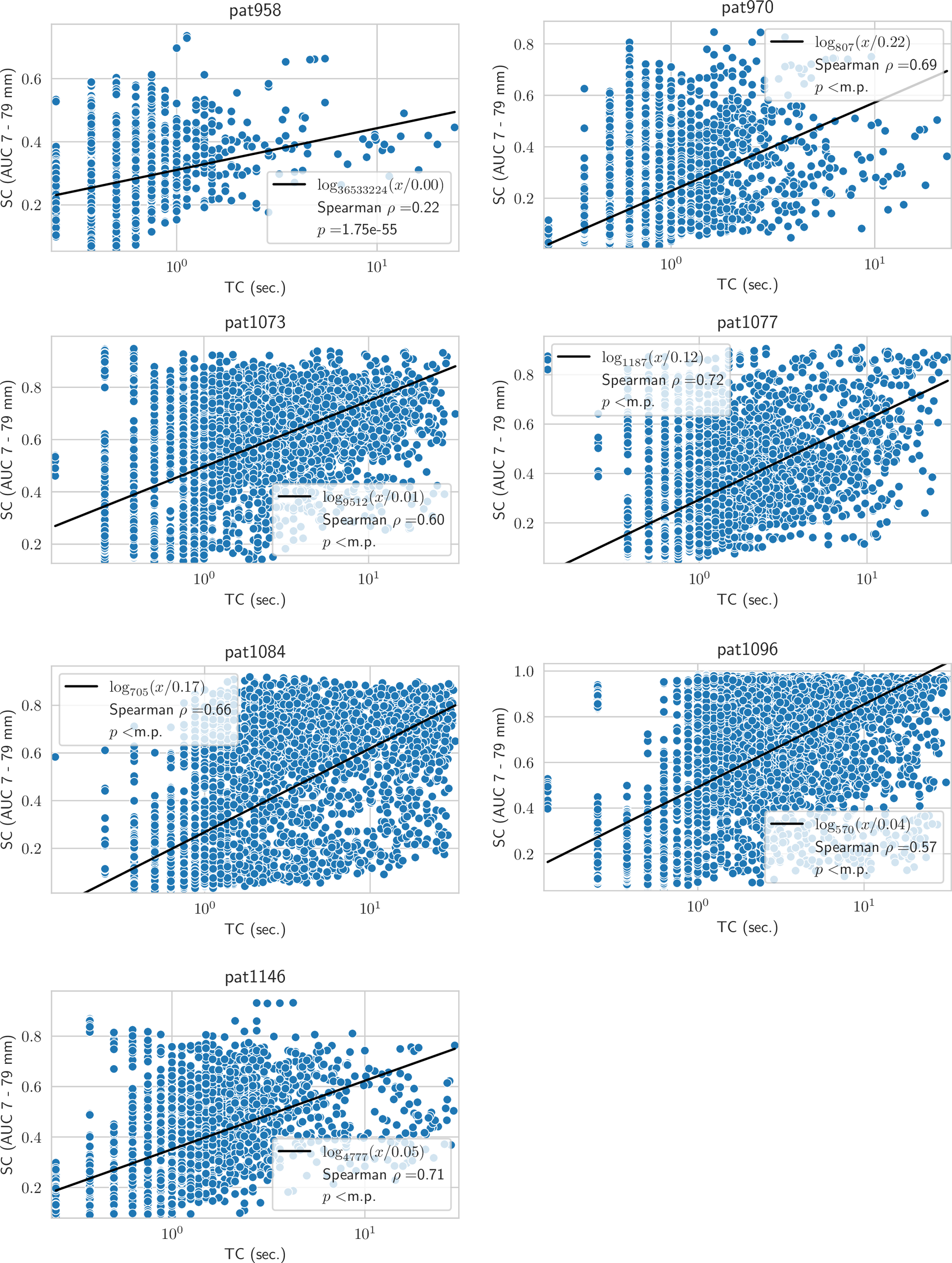

Supplement: S8 Fig — (TIF) [file pcbi.1010919.s011.tif]

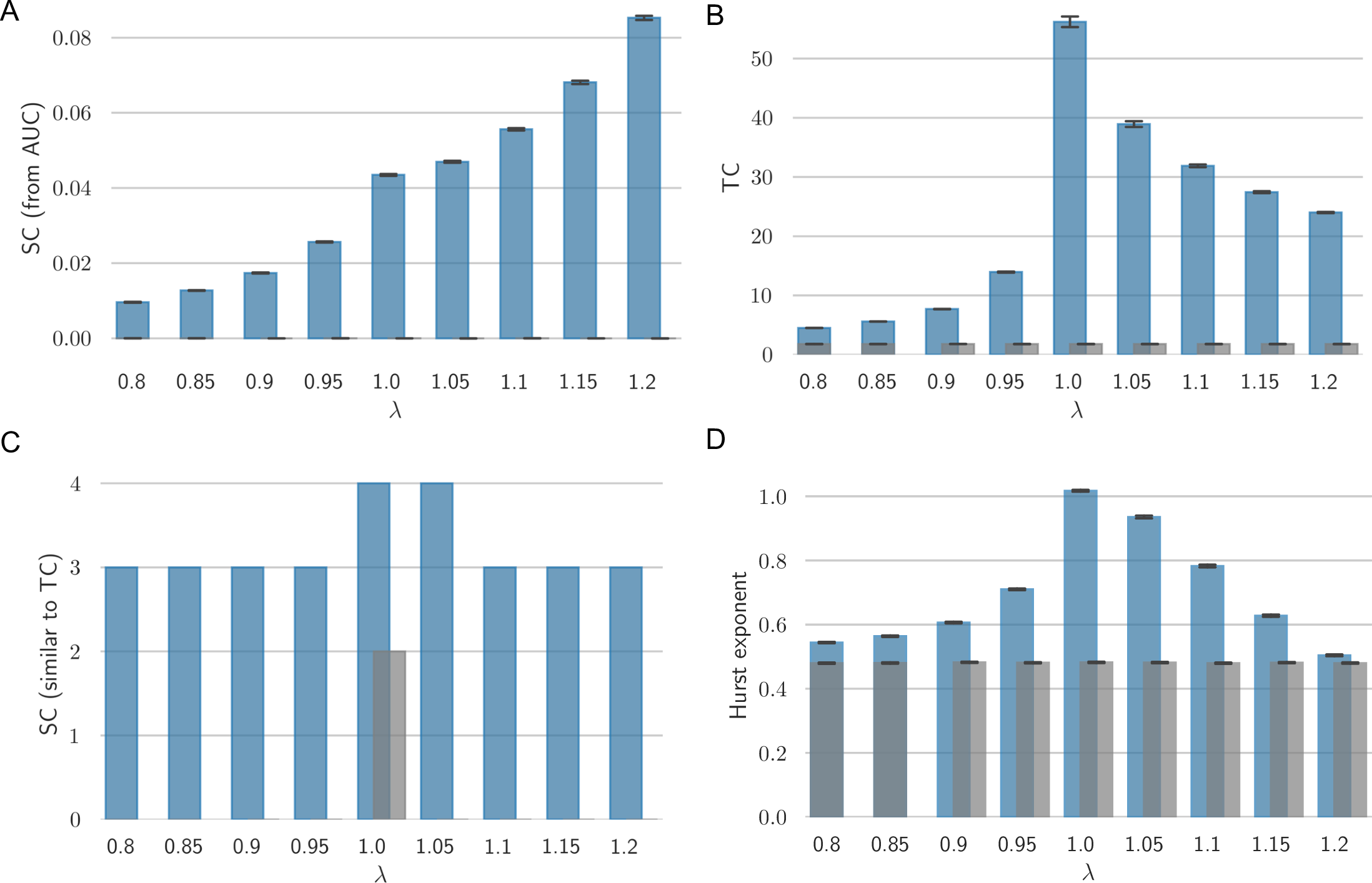

Supplement: S9 Fig — All model parameters are as in the main text of the manuscript (compare Fig 4 in the main text). A Spatial correlations (SC), as defined in the main text of the manuscript (area under the cross-correlation function (AUC)), increase beyond the critical value of λ = 1. B Temporal correlations (TC), as defined in the main text of the manuscript, peak at λ = 1 indicative of a critical point of the model at λ = 1. C Defining SC similar to TC (i.e., first distance of the correlation function to fall below half the value between the value at the first distance and the baseline) shows SC to peak at the critical value of λ = 1. This is only visible in the average (shown here) due to the model being restricted in spatial resolution (only 20 different distances) and single model simulations being noisy leading to non-smooth cross-correlation functions (not shown). Importantly, calculating SC this way is only possible in the model as short interelectrode distances are generally missing in EEG data, which are, however, essential for this quantification (compare Fig 2 in the main text). D The Hurst exponent, as calculated in Hardstone et al., 2014 [26], peaks at λ = 1. This indicates higher self-affinity of the signal the closer it is initiate to criticality. Such self-affinity cannot be observed in the surrogate (time-shuffled) data (grey bars). Hurst exponents were calculated on scales from 100 to 1000 time steps (model simulations were 2000 time steps long). (TIF) [file pcbi.1010919.s012.tif]
