# Supplementary material for: Spatial and temporal correlations in human cortex are inherently linked and predicted by functional hierarchy, vigilance state as well as antiepileptic drug load
Source: PLoS Comput Biol. 2023 Mar 3;19(3):e1010919. doi: 10.1371/journal.pcbi.1010919 (PMC10027224; doi:10.1371/journal.pcbi.1010919)
Supplement: Attachment — Submitted filename: Response_Letter.docx [file pcbi.1010919.s013.docx]

Dear Dr. Taylor,

Dear Dr. Graham,

Thank you very much for considering our manuscript “Spatial and temporal correlations in human cortex are inherently linked and predicted by functional hierarchy, vigilance state as well as antiepileptic drug load” (Manuscript Number: PCOMPBIOL-D-22-01434) for publication and giving us the opportunity to revise our manuscript. We would also like to thank the two reviewers for their constructive and helpful comments which helped us to improve the manuscript further.

In the revised manuscript we have addressed all the comments made by the reviewers. Below we provide a detailed point-by-point response to the reviewers’ comments. Please find a revised manuscript with the changes highlighted in yellow attached to this resubmission along with an unmarked (clean) version of the manuscript.

We hope that the manuscript can now be accepted for publication.

Sincerely,

Christian Meisel, MD

Reviewer #1: In this manuscript by Muller and Meisel, the authors investigate spatial and temporal correlations in data from human patients. They conclude that spatial and temporal correlations are significantly linked, that they are greatest in cortical regions that are highest in the functional hierarchy and that they decline under anti-epileptic drugs. The findings in the data are helpfully replicated by a simple computational model that captures features of criticality. This model shows that spatial and temporal correlations are maximal near the critical point and that they will decline when the excitatory/inhibitory balance is disrupted, as would be expected with anti-epileptic drugs.
It has been known for nearly two decades that long-range temporal correlations may be connected to criticality in the human brain (Linkenkaer-Hansen et al., 2001; the authors cite this). Likewise, there has been work in monkeys, which the authors also cite, showing that temporal correlations are greatest in cortical areas that are highest in the hierarchy (Murray et al., 2014). Similarly, the role of spatial correlations in humans has been previously examined and linked to criticality (Expert et al., 2010; the authors cite this). While these findings have been reported, the connections between them in human subjects has been lacking. The appeal of the manuscript by Muller and Meisel is that all of these disparate results are now brought together in one population of humans and examined for their interrelationships. This made the manuscript very satisfying for me to read and scientifically verified some of the relationships that had been hypothesized by others. Now we know that spatial and temporal correlations are related to each other in humans; previously this had only been suspected. Now we know that the correlations climb within the human cortical hierarchy; we know that anti-epileptic drugs cause them to decline. The paper is clearly written for the most part (see a few suggestions for improvement below) and nicely presented with a wealth of data.

We thank the reviewer for their kind assessment of our manuscript and their discussion of our work within the context of the broader field of research. The comments, questions, and remarks of the reviewer were very helpful, and we are convinced that they helped in strengthening and improving our manuscript significantly. In the following, we give a point-by-point response to the reviewer’s comment and how we addressed each of them in our manuscript.

In what follows, I offer suggestions for the authors’ consideration for possible improvement.
Line 198 and thereabouts: It might be helpful here if you could explain a little more about the model, even though you give references. Specifically, readers may want to know if there are both excitatory and inhibitory neurons and if there are feedback loops and any plasticity mechanisms that could enhance long-range temporal correlations (LRTCs). A sentence or two could tide them over until they dig it up in the supplementary information.

We are very grateful for the reviewer’s comment and have added some sentences in the results section to introduce the model better.

“In short, the model consists of a 2-dimensional lattice of all-to-all connected excitatory and inhibitory neurons whose connection strengths decrease with increasing distance. During a simulation run, all connection strengths are kept constant as we want to explicitly investigate the effects of different connection strengths. The single neurons fire randomly with a certain probability as well as depending on their inputs. Excitatory synaptic inputs increase the likelihood of firing, inhibitory inputs decrease it. Even though self-connections are omitted in the model there are still loops via other neurons that may afford recurrent activity.”

We furthermore expanded on to the model by connecting the mechanisms in the model to the mechanisms of common anti-epileptic drugs.

“Additionally, the neuron network model allows mimicking the effects of AED by either decreasing the excitability of neurons by f_exc, for example via ion channel blockers, or by increasing inhibitory synaptic strength by f_inh, for example via GABAergic drugs. As the results for both mechanisms are qualitatively similar in the model, we only report them for the former (for a direct comparison see also 19).”

Figure 2: Are the colors switched here between plots E, F compared to G, H? I thought orange was high AED in E, F. Yet it is low AED in the histogram plots G, H?

We thank the reviewer very much for pointing out this error. There seems to have been a bug while plotting, and the reviewer is of course right. We have fixed the error so that the orange color now corresponds in both plots to low AED and blue to high AED days.

Figure 3: Could you also look at local spatial correlations within the hierarchy (for example, within OFC compared to within MT)? If so, did those increase also? I realize that such an analysis would have to examine data within a cortical region, and you might not have enough closely-spaced electrodes for that. If that is the case, just say so.

We appreciate the reviewer's comment and interest in such an analysis, which we would have wanted to do as well. Unfortunately, as already suspected by the reviewer, there are not enough closely spaced electrodes within one region and the suggested analysis is not feasible. For example, we have only 4 patients which have more than 5 electrodes in two separate regions, (which are not the same for these 4 patients). Also, please see the interelectrode distance distribution in Figure 2 F which also shows this lack of short inter-electrode distances. We have emphasized this in the manuscript:

“The sparse spatial sampling with only a few electrodes per regions only allowed the evaluation of TC but not SC as a function of functional hierarchy in our data.”

Around line 203: Here you are talking about correlations in the model as it was tuned from the subcritical phase up to the critical point. I noticed that you did not tune the model beyond the critical point into the supercritical region: lambda ranged from {0.9, 0.925, 0.95,0.975, 1}. Did spatio-temporal correlations (STCs) decline again when lambda was increased beyond 1 (supercritical)? Can you show us the other (supercritical) side of the histograms in Figure 4 E, F? If STCs continue to be high with correlations, you could consider switching to covariance (as opposed to just correlation), which should show a decline in the supercritical region (see the Ising model in Beggs and Timme, 2012). The basic idea is that there are times when the correlation will be high but the covariance will be low.

We are thankful for the reviewer’s questions and now provide a new Supplementary Figure S9 showing STC in the model also for values of λ larger than 1. As expected, the TC decrease when λ becomes larger than 1.

Similarly, spatial correlations, when defined in an analogous way, i.e., by quantification of the decay of the cross-correlation function, peak at λ=1, as would be expected at criticality (Fig. S9 C). However, quantifying this decay requires robust estimates of the correlation function at low distances, for which we do not have the data in EEG. Specifically, our SC analysis in EEG is limited (1) by the fixed interelectrode distance and (2) by the low number of electrode pairs and patients in this range (please also see Fig. 2 F).

Thus, already in the previous version of the manuscript and due to the lack of short inter-electrode distances in the EEG data, which would be necessary to quantify the decay robustly, we had opted to quantify SC by the area under the correlation function (Fig. S9 A). Looking at SC and TC collectively thus allows conclusions regarding the positioning of the model (and the data) with respect to criticality: a decrease of both is only possible when moving further towards a subcritical regime. This is also supported by the Hurst exponent peaking at the critical value for λ =1 (please see our response to the reviewer’s next point). To be clearer about this definition of SC, we have added a paragraph in the results section.

“STC increased when λ was increased to the critical value of λ=1 (Figure 4 E, F). As TC decreased again in the supercritical phase (λ>1, Supplementary Figure S9 B), SC kept increasing further (Supplementary Figure S9 A). This can be explained by SC being defined as the area under the cross-correlation function, which generally gets an offset to higher values for increasing λ (data not shown). However, when SC were defined exactly as the TC, i.e., by quantification of the decay of the cross-correlation function, SC also peaked at λ=1, as would be expected at criticality (Fig. S9 C). Quantifying this decay rate, however, requires robust estimates of the correlation function at short distances which were not available in the data due to fixed interelectrode distances and low number of electrode pairs and patients in this range (Fig. 2 F). We thus show SC as measured by the area under the cross-correlation curve for comparability to the data. Thus, for interpretations with respect to criticality, consideration of both SC and TC collectively is preferable.”

In many places you mention temporal correlations, TCs, but you do not use a measure that has been widely adopted in the human literature: the Hurst exponent. Since many other human data papers use it (e.g., Hardstone et al., 2012; Ihlen et al., 2012) it may be good for readers to know how your TCs map onto typical values of the Hurst exponent. I would imagine that it is difficult for your model to produce Hurst exponents much larger than 0.5, as would be expected in a random walk process. As you probably know, this was one of the main shortcomings of the branching model as pointed out by (Poil et al., 2008; which you cite). It is often difficult to get larger values of the Hurst exponent without adding in feedback loops or some type of plasticity in the network. If you do not have H > 0.5, that is fine. But you should say so to clearly delineate the boundaries of your model.

We are thankful for the reviewer’s remark and, upon their suggestion, added the Hurst exponents for our model for varying values of λ in the Supplementary Figure S9 D. We indeed find that our model nicely produces Hurst exponents H close to 1 at the critical value of λ = 1, and decreasing H when looking above and below λ = 1. Additionally, in surrogate model data (i.e., shuffled, thus destroying any temporal correlations), all Hurst exponents were around 0.5, as one would expect for uncorrelated signals. We added a reference to the Hurst exponents in the results:

“This is supported by concomitant changes in the Hurst exponent, a commonly used measure to quantify the self-affinity of a time-series 27, 28. The Hurst exponent peaked at λ=1, where it exhibited values well above 0.5, which would be expected for uncorrelated signals (see Supplementary Figure S9 D).”

And a brief comment in the discussion section:

“Specifically, when tuning the model closer to criticality, dynamics exhibited longer spatio-temporal correlations and thus longer memory within the signal 38. Extended memory (or also self-affinity) in a signal is also often quantified by the Hurst exponent which is 0.5 for uncorrelated signals and grows up to 1 for correlated, self-affine signals. Our model closely matched this expected behavior as it showed Hurst exponents close to 0.5 for simulations far from criticality (λ≠1) and Hurst exponents around 1 at the critical value of the absolute value of the largest eigenvalue (λ=1).”

You mention anti-epileptic drugs (AED) many times and show that they all produce roughly the same effects in terms of causing correlations to decline. I also noticed that you have a table of them in the supplementary material. It might be helpful to include a few sentences for readers who do not specialize in this area of pharmacology, addressing a few questions: Were all AED of the same types or were they different? If different, can you briefly explain how? For example, were they excitatory blockers or inhibitory agonists? Did your model take this into account? Could they have statistically different effects? Did you examine this? I am fine with you summarizing their effects. I just felt unsure if they were all doing roughly the same thing at the mechanistic level, given that there were so many of them, and at widely different dosages. A summary would be helpful to the reader.

We thank the reviewer for this comment. Due to the choice and dosage of AED being determined by the treating physicians, AED and dosage varied across patients and included blockers of excitatory synaptic transmission, inhibitory agonists as well as drugs reducing individual neuron excitability. The variability and still relatively limited size of the dataset limited us from delineating drugs with different mechanisms of action. But we agree that this is an important research question for future research projects. Of note, previous research looking at the different AED mechanisms of action has suggested that, even though the mechanisms of action vary across drugs, their impact on STC may be similar (Meisel, 2020). As suggested by the reviewer, we have added some sentences about this in the results section.

„We compared AED dosages based on the summed prescribed dosage normalized by the defined daily dosage and did not differentiate between different AED due the high variability of AED given across patients (see Supplementary Table S2). Even though different AED have different mechanisms of action, including ion-channel blockers, e.g., Phenytoin, GABAergic drugs, e.g., Clobazam, which increase inhibition, previous work has suggested that their influence on STC may be similar 19.“

Additionally, our model is able to differentiate between increased inhibition and decreased neuron excitability. However, we only report results on the latter as the results are qualitatively similar, further strengthening our reasoning to average over all AED in the data. We added:

“Additionally, the neuron network model allows mimicking the effects of AED by either decreasing the excitability of neurons by f_exc, for example via ion channel blockers, or by increasing inhibitory synaptic strength by f_inh, for example via GABAergic drugs. As the results for both mechanisms are qualitatively similar in the model, we only report them for the former (for a direct comparison see also 19).”

In lines 261-265 in the discussion, you mention the possible confound of an external driving source causing “apparent criticality” in your human data. It is good that you bring this up, as this has been relatively under-appreciated in the criticality community at times. Some very recent work has shown that you can distinguish between criticality caused by external drive from criticality caused by emergent dynamics (Mariani et al., 2022). Relevant for your case, if blockers of synaptic transmission cause signatures of criticality to fade or disappear (as in Meisel, 2020, PNAS, and this paper), then you can largely rule out the hypothesis that the signatures of criticality are caused by a random external drive (Beggs, 2022). Rather, they emerge through the collective interactions of neurons and cortical regions.

Thank you for this important comment and pointing us at these relevant papers. We completely agree with the view of the reviewer and have added a paragraph to set the possibility of an external drive into context, including reference to the studies referenced by the reviewer.

“However, this might seem unlikely based on investigations over 48 hours and the systematic changes observed under AED load or with cortical hierarchy. In particular the ability to tune a system in a way that leads to a collapse of signatures of criticality has been argued to be a strong indicator for critical dynamics emerging from network interactions in the unperturbed state 45,46. In our analysis, the increased AED loads can be regarded as tuning mechanisms of the system, i.e., decreasing the network connectivity, which lead to decreased STC as measures of the distance to criticality.”

References
Mariani, Benedetta, Giorgio Nicoletti, Marta Bisio, Marta Maschietto, Stefano Vassanelli, and Samir Suweis. "Disentangling the critical signatures of neural activity." Scientific Reports 12 (2022): 10770.
Beggs, J. "Addressing skepticism of the critical brain hypothesis." Frontiers in computational neuroscience 16 (2022).

We have included these references in the revised manuscript, thanks for pointing them out.

Reviewer #2: The manuscript presents a highly detailed analysis of temporal and spatial correlations in intracranial EEG data. In general, the methods are rigorous and the manuscript well written. Particular strengths include using 48 hours of recording per subject and analyzing the effect of state of vigilance and medication load.

We thank the reviewer for their review of our manuscript and constructive comments which we believe helped us to further strengthen our manuscript. In the following, we address these comments point by point.

The only significant weakness I noted was the lack of addressing the impact of the number of days since surgery, as the brain can take days to weeks to return to normal after surgery. Drug load is also correlated with time since surgery, as clinicians may initially taper medications down to increase seizure likelihood and then later increase them once enough seizures are recorded.

We are grateful for the reviewer’s comment and agree that this could, in principle, be a confounding effect. Based on the reviewer’s comment, we performed a sub-analysis of the 6 patients who had the lower AED day closer to surgery than the higher AED day. In these patients, we did not observe any significant effect in the opposite direction, which could have suggested time to surgery being the main driving factor based on our data.

Beyond that, we feel that the limited size and setup of our data set does not allow us to delineate the AED effect and the effect of the brain relaxing to its “normal” activity after surgery much further. Thus, we agree that there is further research needed to delineate these effects. Furthermore, we would like to remind that the results we found for SWS and the cortical hierarchy should not be affected by this as there is no timely separation in the data with respect to the surgery. To make the reader aware of this important point brought forward by the reviewer, we added a paragraph in the limitations part of our discussion section:

“For instance, cortical dynamics may potentially take multiple days to relax back to “normal” after surgery for electrode implantation. As the AED load often correlates with the time after surgery this could potentially be another, at least partial contributor to the observed changes in STC under changing levels of AED. In our data, 17 out of 23 patients had their higher medication day closer to the surgery date than the lower medication day. A sub-analysis of the six patients with the low AED day closer to the surgery, however, did not show a significant opposite effect in the other direction, which might have been an indication of the surgery effect. Thus, while the current data does not suggest time to surgery to be a main driving factor, further research is needed to better delineate the effects of AED load and relaxation of cortical dynamics after surgery. Furthermore, these effects should also not affect changes of STC during SWS and along the cortical hierarchy, as these were compared within patients and did not have a timely delay to surgery.”

Minor comments follow:

Results: define gamma frequency range when gamma-power is first mentioned

We thank the reviewer for their comment and added the frequency range after mentioning the broadband $\gamma$-power for the first time in the results.

“Broadband γ-power (56-96 Hz) was used as an index of population firing rate near an electrode 14–18 to derive spatial and temporal correlations and based on prior comparative work across broad frequency ranges 62.”

How do shuffled data with same gamma power

We believe this comment to refer to the surrogate data used as control in our analyses. In short, we first create a time series of the high gamma power which is then shuffled in time (please see also Methods and Fig. 1). This shuffling destroys any temporal and spatial correlation, but maintains the same distribution of values as in the unshuffled data.

“As a result, electrode positions were assigned to a certain region if they fell within 9.5 mm of it” A threshold of 9.5 mm seems a bit too large. Can you provide any data or better justification?

We thank the reviewer for this comment. We think that choosing 9.5mm as a threshold is reasonable for our data for various reasons. First, please note that our primary goal for this spatial assignment is to differentiate different cortical regions along the cortical hierarchy, which in our specific case are many centimeters apart from each other so that even small deviations should not be too problematic. In line with this argument, we generally observe a posterior to frontal trend in the hierarchy of STC which is in agreement with the posterior to frontal hierarchy of the visual pathway. Second, we believe that there are some inherent, methodological inaccuracies associated with the MNI positions and matching them to a template for data such as ours from the Epilepsiae database, and matching them with much higher accuracy to cortical regions may by unfeasible or misleading in the sense that it may suggest an accuracy that is not there. Third, if choosing a much smaller threshold, we would restrict the space too much so that we cannot even approximately cover these regions from the data we have or would be restricted to less electrodes, making the influence of noise relatively larger.

To address this important point, we have transparently stated in the manuscript that it remains a trade-off between spatial accuracy and having enough coverage of the regions in the brain: “which is a trade-off between positional accuracy and maximizing the number of electrodes for each region”

We have added an additional sentence, and hope that this addresses the reviewer’s comment.

“Generally, the regions are far apart allowing for such a relatively coarse assignment and an observation of posterior to frontal changes of our measures.”

“which leads to a non-zero TC measure even in surrogate data” Please define the surrogate data more clearly. How was it created? What exactly did you do with it? This could be an additional strength to the manuscript, but as of now, surrogate data gets a very minimal mention.

We appreciate the reviewer’s remark and have added another subsection and paragraph in the Methods section explaining the surrogate data in more detail and how it may help to rule out finding the results as a result to mere fluctuations in the general power distribution.

“Surrogate Data

As additional controls, SC and TC were also calculated in the same way from randomly time-shuffled power time series (see lower panels in Figure 1). Specifically, the real-data broadband γ-power time series was randomly shuffled within each 2-min segment, thus preserving the same power distribution as in the data but eliminating the temporal correlation of the data. This was done for each electrode separately and, by consequence, eliminated spatial correlations as well. Using these time-shuffled surrogate data, TC and SC were calculated. Comparison of our data results with these surrogate data allowed to rule out that our findings simply stem from fluctuations in the power but were indeed caused by changes in the correlations in space and time.”

“A 2-min segment was finally classified as SWS, if all 30-sec segments in it were classified as SWS.” What was done for the 2-minute segments that only partially contained SWS? Were they included in the non-SWS group or where they ignored?

We are grateful for the reviewer’s comment. Indeed, only 2-min segments which were fully composed as SWS were defined as SWS. All other segments, including those which were only partially SWS were defined as nonSWS. This was done to make sure that we truly captured SWS (in delineation from anything else, i.e., nonSWS). We have added this information to the manuscript:

“A 2-min segment was finally classified as SWS only if all 30-sec segments in it were classified as SWS and as nonSWS otherwise.”
