# Supplementary material for: Spatial and temporal correlations in human cortex are inherently linked and predicted by functional hierarchy, vigilance state as well as antiepileptic drug load
Source: PLoS Comput Biol. 2023 Mar 3;19(3):e1010919. doi: 10.1371/journal.pcbi.1010919 (PMC10027224; doi:10.1371/journal.pcbi.1010919)
Supplement: Attachment — Submitted filename: R2_response_letter.docx [file pcbi.1010919.s014.docx]

Dear Dr. Taylor,

Dear Dr. Graham,

Thank you very much for considering our manuscript “Spatial and temporal correlations in human cortex are inherently linked and predicted by functional hierarchy, vigilance state as well as antiepileptic drug load” (Manuscript Number: PCOMPBIOL-D-22-01434) for publication and giving us the opportunity to revise our manuscript. We thank the two reviewers for their constructive and helpful comments which helped us to improve the manuscript.

In the revised manuscript we have addressed the only remaining minor comment regarding the definition of the surrogate data.

We have also made sure that our manuscript fulfills the requirements of PLoS Computational Biology regarding Figure Files, Data Requirements, Reproducibility and References.

Please find a revised manuscript with the changes highlighted in yellow attached to this resubmission along with an unmarked (clean) version of the manuscript.

Sincerely,

Christian Meisel, MD

Reviewer #1: I think the authors have done a good job of addressing my questions. It is a solid paper, providing important information about humans that we did not know previously.

We thank the reviewer very much for their positive feedback and are happy that we could provide for the reviewer satisfying and meaningful changes and additions.

Reviewer #2: I appreciate the authors attention to the previous comments and their work to improve the manuscript. The manuscript will be an important contribution to this field. I have one remaining concern regarding how the surrogate data was generated. First, I'd like to point out that I greatly appreciate the authors use of surrogate data, which strengthens the paper significantly. I also appreciate the further explanation of the surrogate data generation method which has been added.

We are grateful for the reviewer’s positive assessment of our revised version of the manuscript and added on their concern further down.

However, the explanation of the method brings up an essential question.

It sounds like you have just randomly permuted the time series data. This will not maintain the power distribution as you claim. Reordering the time series points will change the power distribution, which you can easily verify by computing power spectrums before and after the random permutation. Also consider two extreme examples: ordering the points in descending order versus ordering the points based on every other point being descending from the max value or ascending from the min value. One will include only low frequencies, and one will include high frequencies. It is unclear whether your method is valid but the explanation is lacking or whether your chosen method has fundamental flaws.

Standard methods exist for creating surrogate data with a power spectrum that matches given data. On option is IAAFT, Schreiber & Schmitz (1996) [doi:10.1103/PhysRevLett.77.635]. A more robust method is the autoregression/all-pole filter approach used in, e.g., Roehri et al (2017) [doi:10.1371/journal.pone.0174702] and Gliske et al. (2020) [doi:10.1088/1741-2552/abb89b]. I'd recommend using the autoregression/all-pole filter approach.

We thank the reviewer for their comment. We agree that if one shuffles the raw time series before applying the power spectral analysis the power distribution is not preserved. However, within the context of our manuscript, we want to kindly reiterate that this is not how the surrogate data were defined. Specifically, in our manuscript we first perform the power spectral analysis and extract the high gamma power (one value every 0.125 seconds), thus creating a new *power time series* of high gamma power. Only thereafter we shuffle this *power time series* of high gamma power in each 120 second segment. Thus, the distribution of high gamma power does not change as all values for high gamma power are preserved in this time series, but the timely order of these high gamma power values is lost and thus the temporal correlations are eliminated. This approach does not need any further assumptions or coefficient estimation and hence is very robust and computationally efficient.

We apologize for this misunderstanding and have expanded on the explanation of how the surrogate data analysis performed to clarify our approach:

*“Specifically, after extracting the broadband γ-power time series, this power time series was then randomly shuffled within each 2-min segment. As the time series shuffling only happens after the power spectral density is computed the power distribution in the data is preserved but the temporal correlations in the data are eliminated.”*

Thank you for helping us to clarify this more.
